# Arthroscopic surgery for ankle gouty arthritis: a retrospective analysis of clinical outcomes at six month follow-up based on a novel classification system

**DOI:** 10.1007/s00264-023-06057-5

**Published:** 2023-12-15

**Authors:** Baozhou Zhang, Ying Li, Xiaosong Yang, Xiaofeng Gong, Ning Sun, Liangpeng Lai, Wenjing Li, Yong Wu

**Affiliations:** 1 The Fourth Clinical Medical College of Peking University, Beijing, China; 2 Department of Foot and Ankle Surgery, Beijing Jishuitan Hospital, No. 38, Longyu Ring Road, Changping District Beijing, 102208 China

**Keywords:** Ankle, Gouty arthritis, Classification, Arthroscopic surgery

## Abstract

**Purpose:**

This study aimed to evaluate the clinical outcomes, patient-reported outcomes, and recurrence rate of patients diagnosed with ankle gouty arthritis who underwent arthroscopic surgery based on the new classification.

**Methods:**

A total of 51 patients diagnosed with ankle gouty arthritis were included in this retrospective study. A new classification was proposed based on the location and extent of MSU crystal deposition under an arthroscopy view. Patients are classified into different types and underwent arthroscopic surgery accordingly. The primary outcome measure was the American Orthopaedic Foot & Ankle Society (AOFAS) ankle-hindfoot score. The secondary outcomes included the visual analog pain scale (VAS), serum uric acid levels, and the recurrence rate of ankle gouty arthritis at one year postoperatively.

**Results:**

Based on the new classification, five patients were Type I, 24 patients were Type II, five were Type III A, six were Type III B, and 11 were Type IV. The average follow-up time was 23.5 ± 10.9 months. The AOFAS hindfoot-ankle score improved significantly from 70.3 ± 15.9 to 85.6 ± 13.0 (*p* < 0.01). The mean serum uric acid level was significantly decreased from 442.0 ± 109.2 to 540.5 ± 132.4 (*p* < 0.01). The average VAS scale decreased from 3.8 ± 1.9 to 1.4 ± 1.7 (*p* < 0.01). The median of recurrences in one year postoperatively was significantly decreased from 1.5 (1, 3.75) to 0 (0, 0.75) (*p* < 0.01).

**Conclusion:**

A new classification strategy for ankle gouty arthritis based on arthroscopic view was proposed. Patients with ankle gouty arthritis showed significant improvement in ankle function and pain relief after undergoing arthroscopic surgery driven by the new classification.

**Supplementary Information:**

The online version contains supplementary material available at 10.1007/s00264-023-06057-5.

## Introduction

Gouty arthritis, characterised by recurrent self-limiting periarticular pain, is a chronic disease caused by the deposition of Monosodium Urate (MSU) crystals and subsequent inflammatory response. The lower limb, including the foot, ankle, and knee, is often preferentially affected by gout flares [1]. Recent studies have reported an increasing prevalence of gout, ranging from < 1% to 6.8% [2, 3]. Managing gouty arthritis involves suppressing acute flares and treating hyperuricaemia, with long-term urate-lowering therapy being crucial [4–6]. However, a significant number of patients diagnosed with gout do not receive appropriate medication, leading to disease progression [7]. Surgical intervention may be necessary to address MSU crystallisation and deposition.

The advancement of the arthroscopic technique has made arthroscopy a viable approach for the surgical treatment of gouty arthritis. Wang C et al. conducted a study that demonstrated a lower recurrence rate and improved foot and ankle function in patients who underwent arthroscopic removal of gouty crystals from the first metatarsophalangeal (MTP) joint compared to those who received only medication [8]. Similarly, Tang et al. compared the clinical efficacy and recurrence rate of ankle gout arthritis between patients who underwent ankle arthroscopic debridement combined with medication and those who received medication alone [9]. They found that the arthroscopic group exhibited improved clinical efficacy and a lower recurrence rate. Gong et al. showed that combining febuxostat and arthroscopic debridement for MSU crystal deposits effectively lowered uric acid levels and reduced gout flare-ups compared to febuxostat alone [10]. The study involved 61 knees and 38 ankles. Meanwhile, Wang X and his team reported that knee gouty arthritis patients with combined arthroscopic surgery and oral medication exhibited improved knee flexion and extension compared to patients only on oral medication [11]. These studies highlight the advantages of surgical treatments in managing gout.

However, previous studies lacked a comprehensive classification of arthroscopic findings and clear descriptions of corresponding procedures. The classification process has potentially been hampered by the scarcity of pre-existing pertinent studies and the limitations imposed by small sample sizes. Our research, which constitutes the most extensive study in this area to date, is intended to serve as a benchmark for future investigations. In this study, we proposed a new classification strategy based on the arthroscopic view. Patients were classified into four groups based on the location and extent of MSU crystal deposition, and different surgical procedures were employed accordingly. The aim of this study is to evaluate the recovery of foot function in patients diagnosed with ankle gouty arthritis who underwent arthroscopic surgery based on the new classification.

## Materials and methods

### Patients

**Fig. 1:**
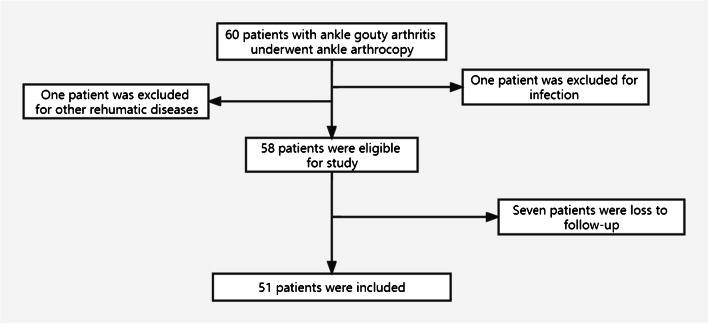
Flowchart of patients’ inclusion

This retrospective study included patients diagnosed with ankle gouty arthritis and who underwent ankle arthroscopy in our department from April 2018 to December 2022. The diagnosis of ankle gouty arthritis was validated according to the 2015 American College of Rheumatology/European League Against Rheumatism classification criteria. We excluded patients presenting with co-existing rheumatic disorders in the foot and ankle, those with foot infections, those who had previously undergone joint sacrifice surgery, and those who were lost to follow-up. The gold standard for diagnosing infection is synovial fluid culture. Synovial fluid cultures were conducted in all patients presenting with febrile symptoms and elevated inflammatory markers, encompassing white blood cell count, erythrocyte sedimentation rate, C-reactive protein, and procalcitonin. An initial pool of 60 patients was considered for the study. Following the application of our exclusion criteria, seven patients were omitted due to loss to follow-up, one due to an existing ankle *Staphylococcus aureus* infection, and one due to an alternate rheumatic disease (Fig. 1). Consequently, the final cohort comprised 51 patients, predominantly male (*n* = 50) with a single female participant (Table 1).

**Table 1:** Demographics of participants

Patient characteristic	Value
Total cohort, N	51
Age, years (mean, SD)	34.1 ± 11.7
Gender (*n*, %)	
Male	50 (98%)
Female	1 (2%)
BMI, kg/m^2^ (mean, SD)	29.9 ± 5.0
Laterality (*n*, %)	
Left	17 (33.3%)
Right	34 (66.7%)
Portal (*n*, %)	
Anterior	34 (66.7%)
Posterior	12 (23.5%)
both	5 (9.8%)
Course, months	48 (12, 84)
Follow-up, months (mean, SD)	23.5 ± 10.9

Data were present as median (interquartile range) for nonnormal variables and frequency (%) for categorical variables. BMI: body mass index

This study obtained ethical approval from the Ethics Committee of our institution (Approval No. K2023-166–00). Verbal informed consent was obtained from all patients prior to their participation in this study.

### Classification

**Fig. 2:**
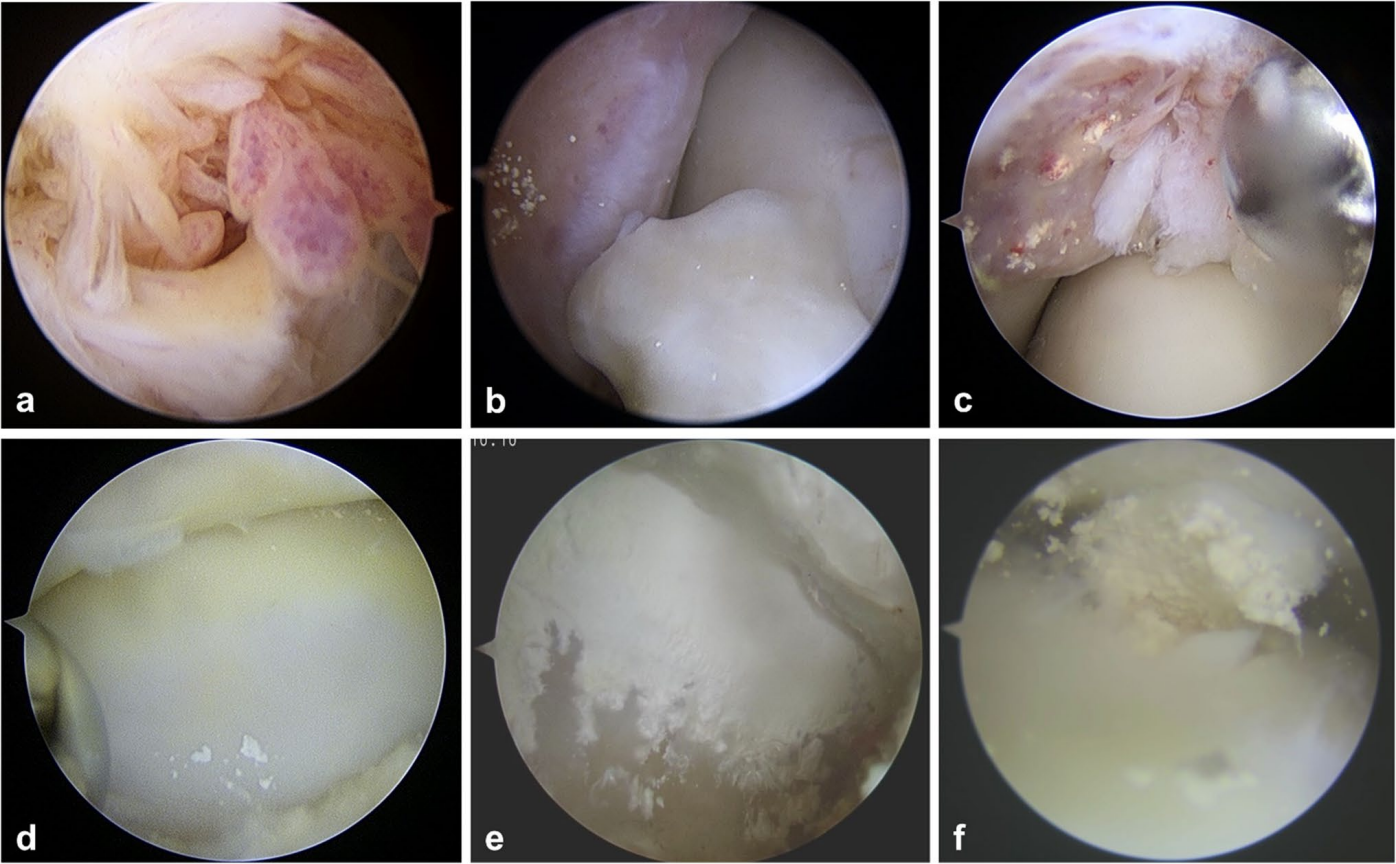
Arthroscopic view of different types of ankle gouty arthritis. (a) Type I, hyperplasia synovial. (b) and (c) Type II, MSU crystal deposition on the anterior ankle and osteophyte in (b). Hyperplasia synovial and MSU crystal deposition on the anterior ankle in (c). (d) Type III A, spot deposition on the surface of the cartilage of the talus. (e) Type III B, sheet deposition on the surface of the cartilage of the talus. (f) Type IV, subchondral MSU crystal deposition

Patients are categorised into four types based on arthroscopic findings. Type I is characterised by the visibility of hyperplasia synovial tissue. Crystalline deposits of MSU may occasionally adhere to this proliferating synovium, yet they spare both bony and cartilaginous structures. Type II is defined by the observable deposition of MSU crystals on bony or soft tissue structures surrounding the joint under arthroscopy, yet the joint cartilage remains unaffected. This typically manifests as crystal deposits along the anterior and posterior edges of the talus, sometimes with osteophyte encrusted with crystals around the joint, and the periarticular ligaments may also incur damage due to crystal adherence. Type III is typified by the arthroscopically visible deposition of MSU crystals on the surface of the articular cartilage. Depending on the extent of crystal deposition on the cartilage surface, Type III is further subdivided into Type III A and Type III B. Type III A describes a scenario where the crystal deposition on the cartilage surface appears spotty, with the longest diameter less than 3 mm, and the total area smaller than 10% of the joint surface. Spot size can be determined arthroscopically by comparison with the 3.5 mm shaver. Type III B is characterised by flaky crystal deposition on the cartilage surface, covering more than 10% of the joint surface. In patients with more severe symptoms, crystal deposits may be observed ubiquitously across the joint surface (video 1). Type IV constitutes a unique classification, described as the combination of a subchondral cyst resulting from an osteochondral lesion of the talus (OLT) and MSU crystal deposition (Fig. 2).

### Operative technique

All procedures were conducted by three experienced surgeons. The surgical portal to arthroscopy involved an anterior approach in 34 cases, a posterior approach in 12 cases, and a combination of both in five cases. The patient was oriented in either a supine or prone position, depending on the surgical portal utilised. In instances where two portals were adopted, a floating position was used. Upon successful spinal anaesthesia, a tourniquet was applied with a pressure maintained between 280 and 300 mmHg. Following the injection of saline into the joint cavity, the traditional medial and lateral portals were established. The inflow of saline assured both a low temperature environment and a clear surgical view. For type I patients, hyperplasic synovial tissues and attached MSU crystals were meticulously excised using a shaver. Supplemental procedures were carried out as dictated by preoperative evaluations and the intraoperative classification of MSU crystal deposition. For type II patients, osteophytes were identified and subsequently debrided. Crystalline deposits on soft tissues were extirpated as much as feasible. Damaged ligaments were repaired as necessitated by the individual patient’s condition. In type III A cases, afflicted cartilage was scraped with a currete (video 2). For type III B patients presenting with small regions of cartilage crystalline deposits, smaller spotty crystalline attachments were selectively removed. However, for those with larger attachments or full joint surface attachments, cleaning was not attempted to avoid excessive damage to the cartilage. For type IV patients, the subchondral lesion was identified with a probe and subjected to debridement (video 3). If the bone cyst is greater than 1 cm in length, osteochondral grafting will be considered.

### Postoperative care

Postoperatively, all patients were advised to commence range-of-motion exercises within 24 h following the surgical procedure. Patients were then placed in a non-weightbearing short leg splint for a period ranging from three to five days. After this period, the splint was replaced with walking boots. All patients were advised to uric acid-lowering medication and lower purine diet after discharge.

Patients were allowed to start bearing full weight at the 1-week mark following surgery. However, for those who had undergone ligament repair, restrictions were imposed, with partial weight-bearing prohibited until six weeks post-surgery.

### Clinical assessments

The American Orthopaedic Foot & Ankle Society (AOFAS) ankle-hindfoot score, visual analogue pain scale (VAS), serum uric acid value, and recurrence rate of ankle gouty arthritis at six months postoperatively were used to evaluate the functional recovery and pain relief postoperatively. The range of motion was recorded preoperatively. The minimum resolution of ROM measurement was 5°. Plantarflexion and dorsiflexion angles were recorded separately.

### Statistical analyses

Statistical analyses were calculated using SPSS software. Normal variables were expressed as means ± standard deviations (SDs). Nonnormal variables were expressed as median (interquartile range [IQR]). Categorical variables were described as frequencies/percentages. Shapiro–Wilk test was applied to determine the data normality. Differences between preoperative group and postoperative group were compared using two-tailed paired Student’s *t* test for normal and Wilcoxon signed rank test for nonnormal variables. A *p* < 0.05 was considered statistically significant.

## Results

A total of 51 patients with a mean follow-up of 23.5 ± 10.9 months were enrolled, 50 (98%) male and one (2%) female. The mean age was 34.1 ± 11.7 years. The mean BMI was 29.9 ± 5.0 kg/m^2^. The median duration of the disease was 48 (12, 84) months. There were 17 (33.3%) cases of left ankle disease and 34 (66.7%) cases of right ankle disease. The anterior approach was used in 34 (66.7%) cases, the posterior approach in 12 (23.5%) cases, and both anterior and posterior approaches in five (9.8%) cases (Table 1).

Based on the new classification, five (9.8%) patients were Type I, 24 (47.1%) patients were Type II, five (9.8%) patients were Type III A, six (11.8%) patients were Type III B, and 11 (21.6%) patients were Type IV (Table 2).

**Table 2:** Classification of ankle gouty arthritis and corresponding surgical procedures

Type	Description	Procedures	*N* (%)
I	Synovial hyperplasia	Synovium removal	5 (9.8)
II	Periarticular MSU crystal deposits	Crystal deposition debridement, osteophyte removal, and ligament repair	24 (47.1)
III	MSU crystal deposits on the cartilage surfaces		
III A	Spotty deposits	Scraping off damaged cartilage	5 (9.8)
III B	flaky deposits	Selective scraping of damaged cartilage	6 (11.8)
IV	Subchondral deposits of the talus	Clearing unstable cartilage and MSU crystal deposits	11 (21.6)
Total ankles			51

The AOFAS hindfoot-ankle score was significantly improved compared with the preoperative assessment (*p* < 0.01). The mean serum uric acid level was significantly lower compared with the preoperative level (442.0 ± 109.2 *v* 540.5 ± 132.4; *p* < 0.01). The average VAS decreased significantly (*p* < 0.01). The number of recurrences in one year postoperatively was significantly lower than the number of attacks per year preoperatively (*p* < 0.01). The mean degrees of the preoperative range of motion was 45.8 ± 19.5°. The median preoperative dorsiflexion was 10 (5, 20) degrees, and the median preoperative plantarflexion was 30 (20, 45) degrees (Table 3).

**Table 3:** Preoperative and postoperative clinical assessment

Clinical assessment	Preoperative	Postoperative	*P* value
Serum uric acid, μmol/L (mean, SD)	540.5 ± 132.4	442.0 ± 109.2	0.000
VAS (mean, SD)	3.8 ± 1.9	1.4 ± 1.7	0.000
AOFAS (mean, SD)	70.3 ± 15.9	85.6 ± 13.0	0.000
RR, n/year	1.5 (1, 3.75)	0 (0, 0.75)	0.000
Range of motion, degrees (mean, SD)	45.8 ± 19.5		
Dorsiflexion, degrees	10 (5, 20)		
Plantarflexion, degrees	30 (20, 45)		

Data were present as mean ± standard deviation for normal variables and median (interquartile range) for nonnormal variables

*VAS*, visual analog pain scale; *AOFAS*, American Orthopaedic Foot & Ankle Society ankle-hindfoot score; *RR*, recurrence rate

## Discussion

This retrospective study analysed postoperative outcomes in patients diagnosed with gouty ankle arthritis who underwent arthroscopic surgery. The findings indicated a substantial reduction in serum uric acid levels and gout attacks within the first year postoperative. Enhanced AOFAS scores and VAS ratings demonstrate functional restoration and pain alleviation in the affected ankle. Additionally, we categorized MSU crystal deposition in the ankle, as observed arthroscopically, to guide procedural decisions.

Our classification primarily focuses on the location of the MSU crystalline deposits, as it dictates the surgical portal and procedure required for each patient. No previous studies have reported an equivalent classification. In light of prior clinical observations and case characteristics, we proposed a novel classification of gouty ankle arthritis. Patients classified as Type II and Type III frequently displayed arthroscopic evidence of synovial hyperplasia and crystalline synovial attachments (a criterion for Type I classification). Type III patients often presented with peripheral crystalline deposits around the ankle (a classification criterion for Type II). This suggests that Types I, II, and III may represent distinct pathological stages of gouty ankle arthritis progression. Consequently, procedures varied based on the respective pathological features. Type IV represents a unique manifestation of ankle gouty arthritis, characterised by crystalline deposits in the bone cysts of OLT. The improved AOFAS scores and VAS underscore the prognostic value of our classification-based surgical procedure.

Compared to preoperative urate values, postoperative values were significantly reduced. It is widely recognised that debridement of MSU crystals can lessen the local burden of urate. However, it remains a topic of debate as to whether the reduction in local uric acid burden can be reflected in serum urate values. Wang and Gong et al. reported a decreased uric acid level in the group receiving arthroscopic intervention, while Wang X documented normal or elevated serum uric acid levels in the arthroscopic group, which included a total of 60 patients with gouty knee arthritis [8, 10, 11]. Our research confirms the former view.

The median recurrence within the first postoperative year was markedly lower than the annual rate of attacks prior to surgery. The deposition of MSU crystals is believed to instigate local inflammation, leading to acute flare-ups of gouty arthritis [12, 13]. Although the precise pathophysiological mechanisms underlying MSU crystal deposition remain elusive, abnormalities in the local structure are known to contribute [14, 15]. Arthroscopic surgery, as a minimally traumatic approach, facilitates the precise removal of MSU crystals and hyperplastic synovium. For patients receiving urate-lowering therapy, the resolution of the monosodium urate crystal burden can span several months to years, even when normal serum urate levels are achieved [16–19]. This period of crystal dissolution harbours the risk for gout flares. Previous studies have demonstrated that arthroscopic treatment can be more effective in curtailing the recurrence rate of gouty arthritis than medication alone [8–10].

Previous studies have demonstrated a correlation between ankle injuries and gout attacks [20–23]. In this study, out of the 40 patients diagnosed with types I, II, and III gouty arthritis, 26 (65%) had a history of prior ankle trauma. The compromised tissue and heightened enzymatic activity could potentially act as nucleation sites for MSU crystal deposition and growth [14]. The removal of this abnormal and damaged tissue may underpin the observed reduction in recurrence rates.

Osteochondral lesions of the talus (OLT) are typically ascribed to either traumatic or haematologic origins, whereas gout-induced OLT is extremely rare [24]. Both Taeho and Tao have each reported a case of a male adolescent presenting with an OLT bone cyst accompanied by MSU crystal deposition [25, 26]. Interestingly, neither patient had a history of trauma to the foot or ankle, and both bone cysts were situated medially to the talus. The current study represents the most extensive sampling of this patient population to date. Among the 11 patients identified with type 4 gouty arthritis, only one (9.1%) reported a history of preoperative ankle trauma. Nine (81.8%) of these 11 patients had bone cysts located on the medial-posterior aspect of the talus, while two (18.2%) had cysts on the medial aspect of the talus. We hypothesise that gout could be a causative factor in the development of OLT, particularly prevalent in the medial-posterior aspect of the talus.

The primary limitation in ankle mobility noted in our study was dorsiflexion, and we observed a higher frequency of anterior portal usage. This suggests that gouty arthritis predominantly affects the anterior region of the ankle.

Our study has a few limitations worth noting. First, it lacks a golden standard therapy control group. This makes it difficult to compare the effects of arthroscopic intervention against a medication-only approach in a controlled manner. Second, the small sample size and heterogenous cohort limit the generalisability of our findings and conclusions. And the efficacy of each subtype cannot be discussed separately due to the small sample size. Future studies with larger patient cohorts are needed to validate our results. Lastly, the follow-up period of our study was relatively short. For a more comprehensive understanding of the long-term efficacy and potential benefits of the arthroscopic procedures, a study with a more extended follow-up period would be beneficial.

## Conclusion

In this study with a mean follow-up of two years, patients with gouty arthritis of the ankle showed significant improvement in ankle function and pain relief after undergoing surgery based on a new classification. Additionally, there was a marked decrease in serum uric acid levels and gout recurrence within the first year postoperative.

### Supplementary Information

Below is the link to the electronic supplementary material.

Supplementary file1 (MP4 20775 KB)

Supplementary file2 (MP4 31058 KB)

Supplementary file3 (MP4 25955 KB)

## Data Availability

The data that support the findings of this study are available from the corresponding author upon reasonable request.
